# Social Hostility in Soccer and Beyond

**DOI:** 10.1371/journal.pone.0153577

**Published:** 2016-04-14

**Authors:** Niels J. Van Doesum, Jan-Willem Van Prooijen, Lot Verburgh, Paul A. M. Van Lange

**Affiliations:** 1 Department of Experimental and Applied Psychology, Vrije Universiteit Amsterdam, Amsterdam, The Netherlands; 2 Department of Clinical Neuropsychology, Vrije Universiteit Amsterdam, Amsterdam, The Netherlands; Philipps University Marburg, GERMANY

## Abstract

Social hostility is seldom expressed overtly. More often than not, individuals try to get their hostile message across without risking violent altercations. However, subtle and relatively covert hostility is not easy to research. We suggest a novel way with the SoMi paradigm, a social decision making task that offers participants the opportunity to be socially mindful or socially hostile by leaving or limiting choice to others. Sampling a general population we find that, relative to friends and strangers, foes are indeed met with greater social hostility (Study 1). Focusing on the highly competitive environment of youth soccer, we find that rival team members elicit social hostility, whereas teammates elicit social mindfulness (Study 2). We conclude that social mindfulness and social hostility play a subtle role in the dynamics of interpersonal and/or intergroup relationships, in which leaving or limiting choice is one of the subtle ways to express benevolent versus hostile intentions; the SoMi paradigm may thus be helpful in identifying which way the ball rolls.

## Introduction

In any competitive environment, social hostility is a common phenomenon. Consider the case of soccer. To many, soccer is more than just a game. Deeply ingrained in almost any level of play lies the urge to win, to gain prestige, to establish dominance; it is us against them and, ultimately, against the world. “Soccer is war,” the late Dutch coach Rinus Michels—allegedly—said; “if you’re too nice, you’re lost” [1]. Little did he know how true his observation may still ring. During the 2014 World Cup, for example, Uruguay vedette Luis Suarez sank his teeth into one of his Italian competitors’ necks; a quick internet search confirms that this is far from the only instance of aggressive conduct and soccer brawls. High performance may be a factor in such enhanced aggression [2], or simple participation and interest in high contact sports [3,4], which soccer undeniably is. In the competitive context of a team sport like soccer, ingroup favoritism will be readily accompanied by outgroup derogation, often leading to various degrees of antagonism and hostility. Such hostility can be instrumental in forming and maintaining a social identity [5], and stereotypes of perceived coldness and feared competence may conceivably underlie such sentiments [6,7]. However, expressing these sentiments does not always have to result in brash actions like biting competitors or other bellicose behavior. Communicating social hostility can also be achieved in much more subtle ways, be it in soccer or other antagonism-prone situations.

At first glance, subtleness and hostility are not likely companions. Defined as a negative attitude that consists of enmity, denigration, and ill will towards others [8], hostility is much sooner associated with open conflict and explicit aggressive behavior. But in actuality, not every hostile impulse will lead to clear and overt aggression; that would not align with the highly civilized societies most people currently live in [9]. There must be more hostility felt than reaches the surface, then, and repression and/or inhibition is not always the best answer at an individual psychological level. To examine covertly conveyed hostility—the kind that may just scratch the surface, present but not dominantly so—is not easy, however. Extant behavioral measures typically target clear displays of aggression that have direct negative consequences for others, like administering hot sauce or noise blasts [10–12]. Because such measures focus on the extent to which people are willing to let others suffer physically (i.e., to hurt them), they are not well suited to examining the numerous interactions in which people feel the urge to communicate social hostility without the need, wish, or opportunity to actually hurt others; in other words, to start a conflict. Yet such communications are a vital part of human interaction. For example, we may block useful information from reaching disliked others, or we may remove options before they can choose—like taking the last available macaroon from a plate of cookies.

Such behavior is actually well captured in the SoMi paradigm, recently developed to assess social mindfulness. This measure hinges on leaving or limiting other people’s choices, and thus their control over situational outcomes [13,14]. Leaving choice can be construed as prosocial, because most people appreciate having control [15–18]. By the same token, limiting choice leaves a message of disassociation that is easily picked up. For example, it only takes two subsequent instances of limiting choice for someone to be less trusted and liked, to be a less desirable collaborator, and to be seen as more self-oriented; in short, to be seen as less interested in others ([13], Study 2a-b). But if that is how unmindful actions are perceived, then maybe this kind of behavior can also be used strategically to express hostile motivations; and if such behaviors are indeed used to distinguish between friend and foe, then this would offer a good way into examining social hostility without having to resort to measures of intended physical pain.

To ensure a certain level of animosity in these preliminary studies on social hostility as measured with the SoMi paradigm, we focused on two strong situations featuring stereotypical friends and foes. Stereotypes per se do not necessarily drive behavior, however, but rather the corresponding emotions [6]. The well-known BIAS map of stereotypes [6,7] suggests that between the fundamental dimensions of warmth and competence lie admiration, contempt, envy, and pity; these emotions may all come into play when deciding to leave or limit choice to others. For example, some findings have suggested that contempt inhibits both active and passive facilitation tendencies, pity inhibits passive facilitation, and admiration inhibits passive harm ([6], p. 637). Applied to the various targets in our studies, such emotions are likely to influence participants’ decisions in the SoMi paradigm.

### Mild Levels of Social Hostility

To be socially mindful is to be thoughtful of others in the present moment and to consider their needs and wishes before making a decision [14]. This can be operationalized as “making other-regarding choices involving both skill and will to act mindfully toward other people’s control over outcomes” ([13], p. 86). Skill and will imply that a combination of ability (i.e., perspective taking) and motivation (e.g., empathy or other benevolent motivations) can result in prosocial behavior. But perspective taking may also backfire by intensifying feelings of competition and promoting unethical behavior [19]. Social hostility is thus composed of the same skill, but combined with ill will towards others.

Methodologically, social mindfulness is defined in terms of *leaving* choice for others, social hostility in terms of *removing* this, and the tendency to do neither could be labeled as ‘indifference’ [14]. Social mindfulness and social hostility can therefore be measured as opposite ends of a continuum. Conceptually, however, it is much more plausible that both concepts do not operate in concert and are typically activated through different interpersonal mechanisms. For example, social mindfulness is more likely to operate as a default for approaching friends—and even strangers—than social hostility. Rather than direct opposites, they may be activated by mechanisms that are quite distinct and only remotely related. Empathy might activate social mindfulness, for instance, whereas rivalry might activate social hostility. Comparable to the concepts of trust and distrust [20–22], social mindfulness and social hostility lead their own lives in many situations and will hardly ever be activated by mechanisms that should be conceptualized as two extremes on the same continuum.

As an example, imagine that Chris and John, who just finished a match of soccer, enter the club canteen and want to have a bite with their beer. The bar is running out of food, however, and there are only three cheese sandwiches left, next to a single hamburger. Chris makes it to the counter first. If he wants to be nice, he will order a cheese sandwich, so John can still choose between a burger and a sandwich. If John is his teammate, Chris will probably do so—friends deserve a choice. If John is a competitor, however, and maybe even on the winning team, Chris may very well go ahead and claim the burger for himself, making his socially hostile point in a subtle manner by limiting his rival’s choice.

Based on similar decisions, can the SoMi paradigm be used to detect mild levels of social hostility? In two studies, we test if people indeed classify friend or foe by leaving or limiting the choice of their interaction partners when choosing from a common set that contains one unique product (e.g., one green and three red apples). We target situations in which participants can be expected to have an undisputed qualm about certain interaction partners as compared to others, and a good reason to express some hostility. Sampling from a general population, in Study 1 we thus examine whether participants limit options more often for someone they truly dislike (foes), relative to strangers (i.e., by taking the unique option). Study 1 also examines whether participants leave more choice to someone they like (friends), relative to strangers. In Study 2 we examine whether social hostility might also be activated in naturally occurring competitive contexts. As a globally appreciated and practiced team sport, soccer provides one of the most pervasive instances of such inherent competition. We thus specifically investigate whether young male soccer players exhibit tendencies toward limiting options for players of a rival club.

## Methods and Results

### Ethics Statement

All studies were reviewed and approved by the Scientific and Ethical Review Board (VCWE) of the Faculty of Behavioral & Movement Sciences, VU Amsterdam. Participants provided written informed consent prior to participation. For minors, this was also obtained from their parents or legal guardians.

### Study 1: Classifying Friend or Foe

#### Participants and design

Study 1 was an online study conducted on Amazon’s Mechanical Turk. To generate sufficient power for an a priori unknown effect size, we set a minimum of 250 participants, and finished data collection after receiving full responses from 273 subjects. Three conditions were randomly assigned in which the target was either to be a friend, a stranger, or a foe. In a strict manipulation check at the end of the procedure, 21 participants did not report back correctly what relation they were instructed to have to the target. We chose a conservative approach in which we excluded these subjects from analyses. The final sample thus consisted of 252 participants (134 men) between the ages of 18 and 70 (*M*_age_ = 33.10, *SD* = 10.70). Of those, 85% reported to be White/Caucasian, 8% African-American, 2% Hispanic, 4% Asian, and less than 1% Native American or ‘other’. Race did not influence the results. Further, we ran some exploratory measures and related analyses that did not influence conclusions regarding the current research question; for the interested reader, these are described in S1 Text.

#### The SoMi paradigm

In Study 1 we developed the dyadic SoMi paradigm that was introduced in previous research on social mindfulness [13]. As the first of two people, participants were asked to choose a product from various products that were shown onscreen. They were instructed that a chosen item would not be replaced, so that their (imagined) interaction partner could not also have that item. Per experimental trial the products were identical, except for one that was slightly different. The ratios of the available items varied between one unique versus two identical items, or one unique versus three identical items (e.g., one green and three red apples, or one gold colored versus two green colored gift boxes). There were 12 such trials in total, each using different kinds of products. Choosing the identical product would leave the other person a choice, and was scored as socially mindful (1); taking the unique product would limit the other person’s choice, and was scored as socially unmindful (0). The final score was the proportion of socially mindful choices across all experimental trials. This constituted our dependent variable.

**Fig 1:**
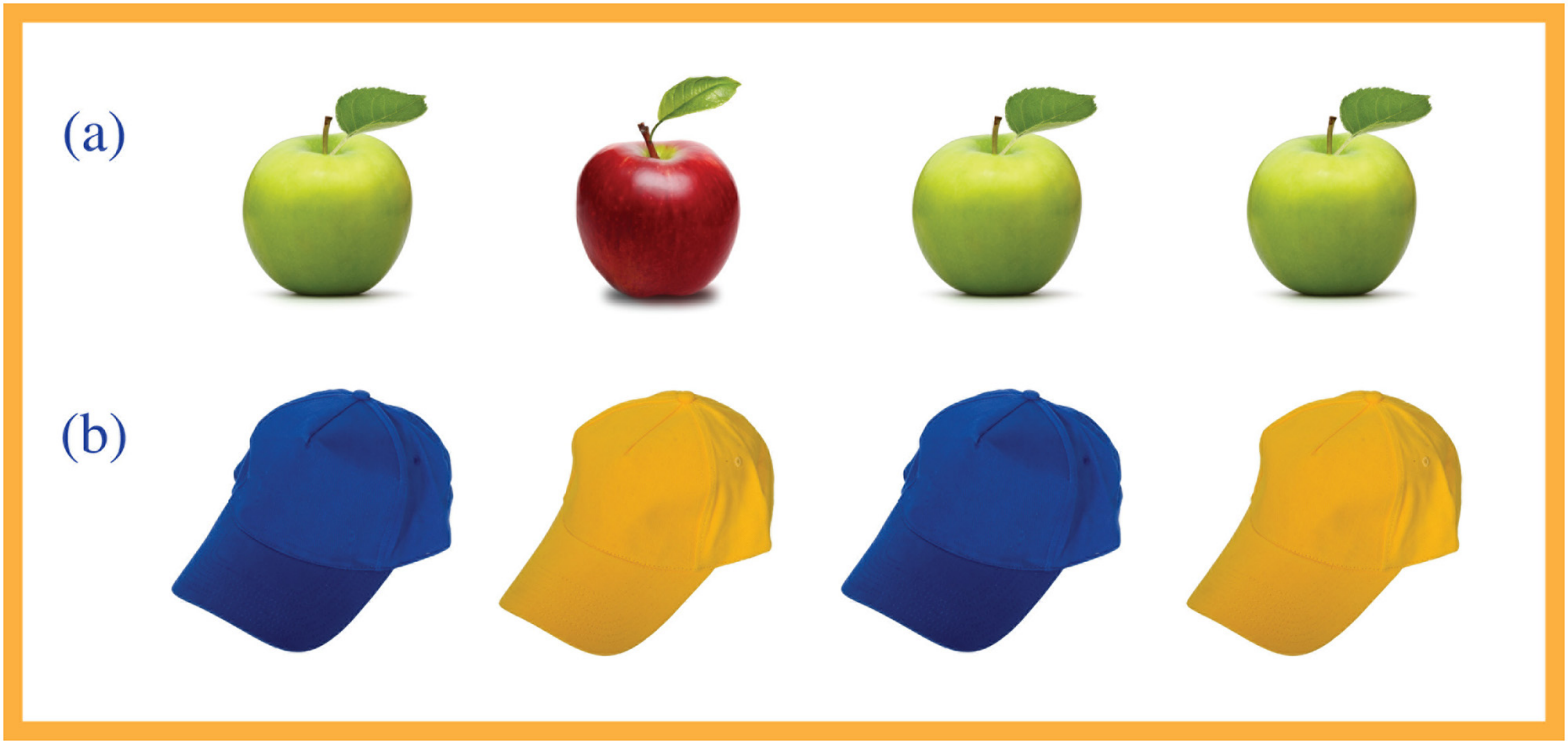
Example of an experimental (a) and a control trial (b) in the SoMi paradigm.

To these experimental trials we added 12 control trials in which the participant’s decisions were socially inconsequential. Control trials offered two versus two (in case of four items in a trial) or three identical products (when three items), and were included to counter habituation while performing the task. Decisions in these trials had no real consequences for the other, and thus were not included in computing the final score. All 24 trials were offered in fully randomized order, with the products randomly placed on a horizontal line onscreen. See Fig 1 for an example of an experimental and a control trial.

#### Procedure

In the introduction to the SoMi paradigm, all participants read: “The task you are about to perform involves two people; you and someone else.” Participants in the friend condition then read: “Imagine that the other person is a close friend of yours, and you experience some true feelings of liking even when only thinking of this friend.” In the stranger condition, participants read: “Imagine that the other person is someone you haven't met before, and will not knowingly meet again in the future (because you and the other will not get to know or see each other in person). You have no specific reasons to like or dislike this person.” And finally, participants in the foe condition read: “Imagine that the other person is someone you have a strong and longstanding conflict with, and you experience some true feelings of disliking even when only thinking of this person.”

#### Results

**Fig 2:**
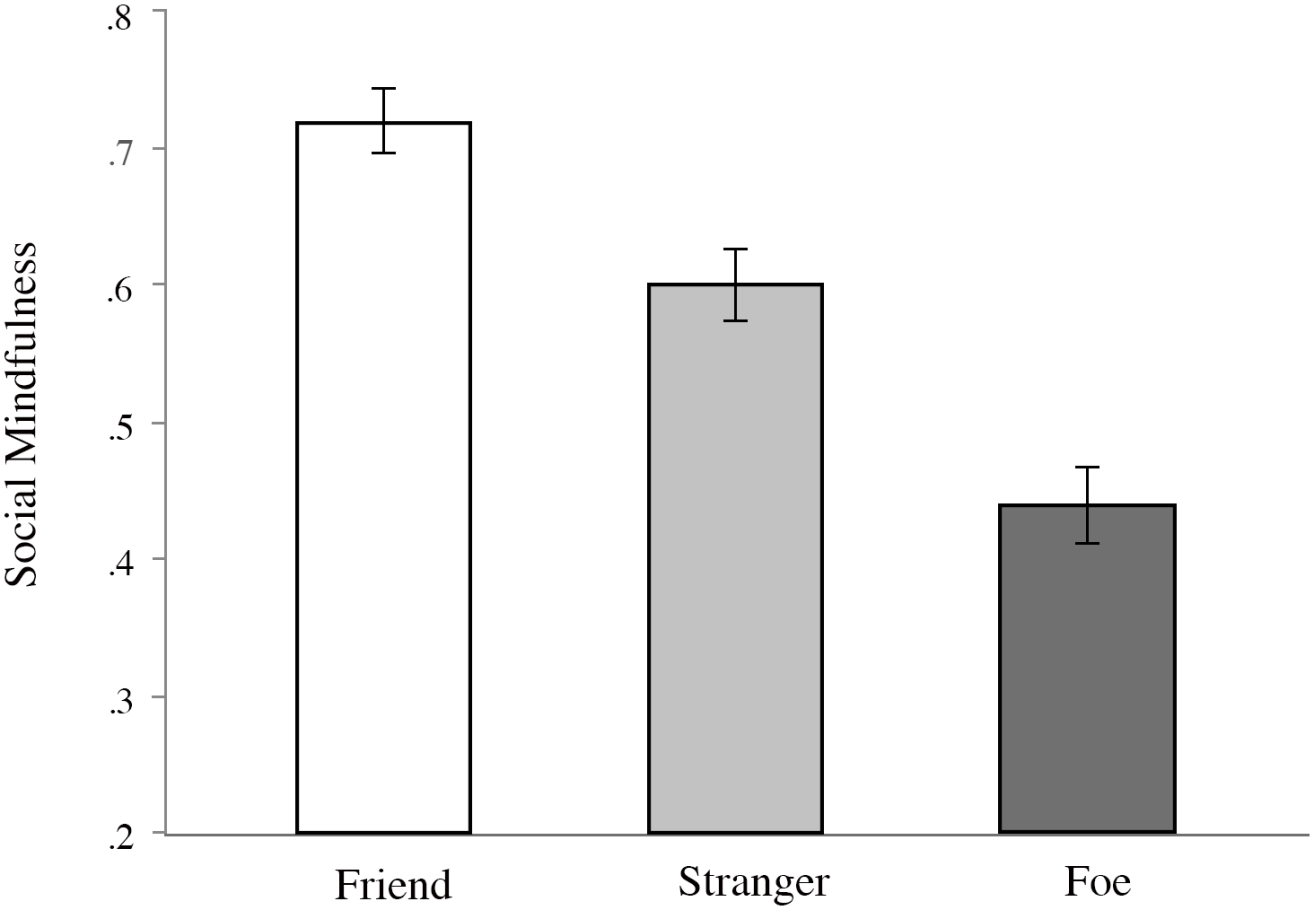
Mean scores on social mindfulness per condition in Study 1. Error bars represent standard error.

Preliminary linear regression analysis showed an age effect; older participants were slightly more socially mindful in general, *b* = 0.005, *t*(250) = 3.17, *p* = .002, CI_95%_ [0.002, 0.008]. We therefore controlled for age in a general linear model pitting condition (friend, stranger, foe) against social mindfulness. Condition revealed the predicted main effect on social mindfulness, *F*(2, 248) = 28.45, *p* < .001, η_p_^2^ = .19. With scores regarding strangers seen as a neutral baseline (*M*_stranger_ = .60, *SD* = .24), participants were indeed more socially mindful to their friends (*M*_friend_ = .72, *SD* = .22) and socially hostile towards foes (*M*_foe_ = .44, *SD* = .24). Pairwise comparisons showed all differences to be significant at the *p* < .01 level; see Fig 2 for a visualization.

### Study 2: Soccer and Social Hostility

#### Participants and design

From the subject pool in a larger research project [23], 135 young male soccer players participated in this group level study on social mindfulness and social hostility. The soccer setting of this study was instrumental in anticipated levels of competitive intergroup motivations. Given the observed effect sizes in Study 1, we estimated this sample to be large enough to capture small to medium effects. The majority of the participants, 102 in total, followed the talent development program in one of three professional soccer clubs within the Dutch “Eredivisie” (premier league), and actively played in a soccer competition at the highest possible level for their age. The other 33 participants were amateur players who trained and played for a soccer club in a lower ranking (amateur) competition in the Netherlands. Ages ranged from 7–18 years, *M*_age_ = 12.43, *SD* = 2.57.

Most of the measures in the overarching research project focused on executive functioning, and were theoretically unrelated to our hypotheses. Here we only report on what was relevant to testing social mindfulness and social hostility in an ingroup-outgroup context in the realistic and highly competitive setting of (elite) soccer competition. In a mixed within-between subjects design, social hostility was assessed in two separate rounds. The first round was played with a stranger in mind, to be used as baseline. In the second round, participants imagined either a teammate (ingroup) or a rival team member (outgroup) as the target.

#### SoMi paradigm

For this study the original SoMi paradigm with five different product categories [13] was expanded to include two additional categories. To increase ingroup identification, we added items that were specific to the various clubs and reflected their identity, like memorial long-drink glasses, specific brands of soccer balls, or shawls with the club logo, all in two different designs. Two of the seven categories of products were ingroup-specific, and five remained general (separate analyses without the scores on the two ingroup-specific items did not alter the conclusions; see S1 Text). Scores were based on 14 trials per round.

#### Procedure

Data were collected in quiet and/or private rooms at the training facilities of the participating professional or amateur clubs. Our brief study on social mindfulness and social hostility was embedded in a longer procedure that took about 1.5 hours to complete [23]. This procedure required the experimenters to be present in the room, who were trained to use standardized instructions and to keep their behavior neutral, equal, and unobtrusive at all times. Measures were taken in set order and in single sessions per participant.

Upon starting the task, participants were instructed to imagine they were performing this task together with someone else. It was emphasized that they were always the first to choose, and that items would not be replaced. After finishing an example trial, participants read: “This round you play with someone you don’t know.” Immediately after finishing the first round, a second round was played in which the participants were randomly assigned to an ingroup and an outgroup condition. In the ingroup condition, participants were told that the other person now was “someone from your own team.” In the outgroup condition, the other was said to be “someone from another team, for example …” Here we inserted the name of the club that traditionally had the strongest rivalry with the participant’s own club.

#### Results

**Fig 3:**
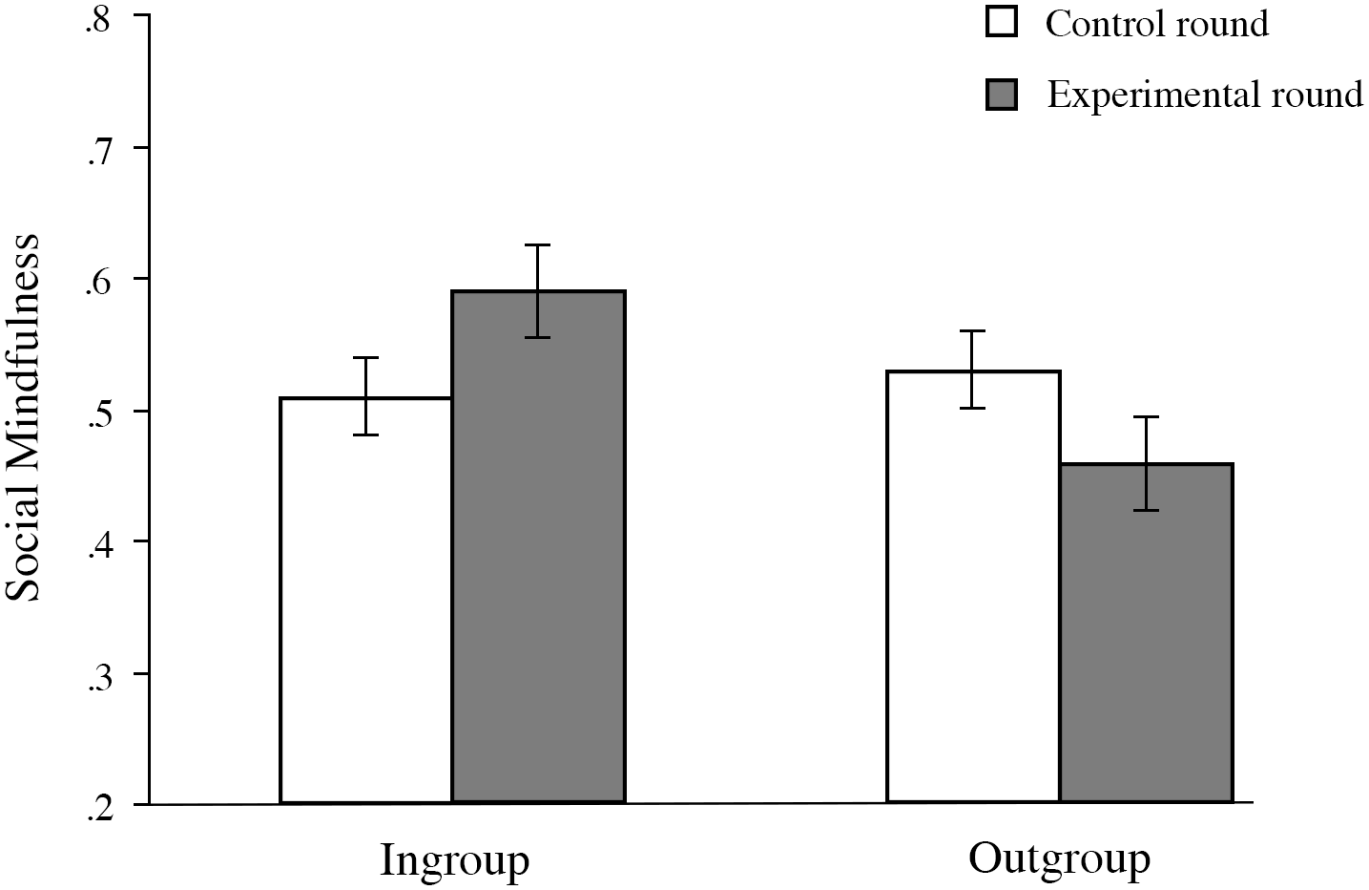
Mean scores on social mindfulness in Study 2. Error bars represent standard error.

A preliminary check did not reveal any age related differences on social mindfulness (*p* = .537), which is understandable given that variation in age was small. Hence, we did not control for age in the final analysis. The overall mean score on social mindfulness in round one (control) was .52 (*SD* = .20). We used a repeated measures approach with control versus experimental round (i.e., rounds 1 and 2) as within-participant variable, and experimental condition (ingroup, outgroup) and affiliation (professional, amateur) as between-participants variable. While there was no main effect for control versus experimental round, *F*(1, 131) = 0.59, *p* = .443, the interaction between condition (ingroup, outgroup) and the two rounds of the SoMi paradigm proved statistically significant, *F*(1, 131) = 21.62, *p* < .001, η_p_^2^ = .14. In the experimental (second) round ingroup members (*M*_ingroup_ = .59, *SD* = .26) exhibited greater social mindfulness towards one another than outgroup members (*M*_outgroup_ = .46, *SD* = .23), while both significantly differed from their corresponding baseline scores regarding strangers in opposite directions (higher for ingroup, lower for outgroup), *F*(1, 131) = 7.71, *p* = .006, η_p_^2^ = .06, and *F*(1, 131) = 14.35, *p* < .001, η_p_^2^ = .10, respectively. In other words, while teammates elicited social mindfulness, rivals elicited social hostility (Fig 3). The same analysis showed no interaction between round and affiliation (professional, amateur) in scores on social mindfulness, *F*(1, 131) = 2.41, *p* = .123, η_p_^2^ = .02, revealing no difference between elite and amateur players.

## Discussion

Short of hurting or yelling at them, how do people deal with others they do not like in regular, everyday interactions? And how can mild hostility be communicated without directly risking a situation spiraling into conflict? Various verbal and nonverbal ways are conceivable, active or passive, and each of them is more or less effective. One way we found to be effective involves the novel construct of social mindfulness, but then in a hostile form as shown in decisions that are consistently *unmindful*. Sampling both a regular population and a more specific group of competitive young soccer players, we show how participants clearly marked the competition by limiting opponents’ choices in shared situations, while leaving choices for friends and teammates. This provides initial support for the notion that social hostility can be expressed—and measured—in simple behaviors like taking away a unique item from a common set to be shared. Where expressions of social mindfulness may smoothen friendly interactions, the clear but subtle communications of social hostility seem to be used to ‘draw a line in the sand’; positions are taken without being too offensive. This makes the SoMi paradigm a good, practical, and unobtrusive measure of social hostility.

Paradoxically, the SoMi paradigm was initially developed to assess prosocial behavior [13]. The paradigm stands out from other psychological measures like social value orientation (SVO) [24–26], decisions in a variety of economic games and social dilemmas [27], or behavioral measures like charitable giving [28,29] and volunteering [30] by the fact that socially mindful decisions do not always require much sacrifice to self-interest; it can be almost cost-free. This makes the observation of other-regarding decisions more important than a tally of what everyone receives at the end. In social mindfulness, simply acknowledging others is more important than the situational outcome; and more than anything else, it is the thought that counts [14]. In actuality, though, this thought can have many colors, including benevolence, indifference, and hostility.

Whether reflecting prosociality or social hostility, the mechanism underlying the direct social consequences in the SoMi paradigm can be found in leaving or limiting choice to others, and thus in providing or precluding a sense of control and agency [31]. As a manifestation of benevolent intentions, leaving choice is indeed a quick way of communicating that others are granted voice in the outcome of a shared situation (for the benefits of having voice, see [32,33]). Openly frustrating choice is denying interaction partners this voice, this desirable feeling of control over the outcome of the situation. Knowing that it would have been little effort, being denied this sense of control (i.e., being treated unmindfully) then can be read as “you are not even willing to do *that* for me; that is how unimportant I must be to you.” Previous findings suggest this is indeed the case: just one repetition of behavior perceived as unmindful can be enough to construe someone as self-centered and less trustworthy [13]. Here we turn this reasoning around and provide preliminary evidence that people may use this kind of behavior to actively distinguish between those they like and those they do not.

### Aggression versus Hostility

In the context of the SoMi paradigm, it is important to distinguish our use of social hostility from what is commonly known and understood as aggression. Aggression is behavior directed toward another individual with the immediate, proximate intention to cause harm, with the target motivated to avoid such harm [34,35]; social hostility as discussed here does no such thing. Limiting someone’s choice will not immediately harm the other in any physical or even direct psychological way. Still, it sends a reasonable message of antagonism, maximally to be understood as a warning that more could be in store. A bark rather than a bite, open conflict and willful damage are not the object. Social hostility is thus more likely to involve proximal goals of communicating scorn, irritation, or derision, for example. In the absence of behavior that is openly harmful or intended as such, it cannot be categorized as truly aggressive behavior. Still we argue that consistently limiting someone’s choice can effectively express underlying aggressive affects [36].

Generally speaking, hostility contains a cognitive, an affective, and a behavioral component [37]. Like being aware of potentially harmful effects in extant aggression measures, cognition is represented in the SoMi paradigm by the actor’s ability to see the social consequences of leaving versus limiting choice. Behavior is expressed somewhat differently, however, in that limiting choice is hardly painful or harmful to others, creating a lower threshold for hostile cues than hot sauce, noise blasts, electric shocks [38], or other variations on that theme. It is also not as manifestly aggressive as sticking needles in a voodoo doll that represents a specific target [39]. Compared to such measures, the SoMi paradigm differs in two important ways: (1) the lower threshold makes it easier for participants to accord response with affect; at the very least, the SoMi paradigm targets milder kinds of hostile affect—it is expressing subtle hostility without expressing aggressiveness or causing pain, even hypothetically; (2) the social cue of limiting choice leaves reasonable room for interpretation [40]: When inadvertently confronted, it is always possible to hide behind the veil of ignorance by claiming that you did not realize you were being hostile, or that you really wanted that unique item. This is much harder to do with quantities of hot sauce or high levels of noise. However, to what extent differences are gradual, qualitative, or a combination thereof is a question for future research. If anything, we would expect to find moderate correlations in support of the latter [37].

### Contributions

How do our preliminary findings contribute to the literature on hostility? For one thing, we have shown that the SoMi paradigm promises to be a clear, unobtrusive and practical way to assess mild levels of social hostility. Building on the finding that consistent unmindful choices elicit slightly adverse reactions to the actor [13], this level of unmindfulness may also be recruited to accentuate existing or arising antagonism—and the current studies suggest it will. But more importantly, mild hostility that barely breaches the surface is an area of research that has not received much attention yet, even though it is conceivably more widespread than the open aggressive altercations that are the focus of a lot of extant research, with more societal impact. The SoMi paradigm may be helpful in unlocking this important yet under-researched field.

One of the reasons it can help is that the behavioral yet hypothetical choices in the SoMi paradigm are conveniently wedged between existing self-/other-report measures [41–45] or scenario studies [46] with their traditional issues, and laborious behavioral measures of hostile aggression [10–12] that come with their own validity challenges (see, e.g., [47]). Further, the SoMi paradigm does not rely on language or textual understanding much, and has proven relationships with prosocial concepts like SVO [48,49], empathic concern and perspective taking [50], and other-orientations [13,14]. And while ‘minding others’ as targeted in social mindfulness initially may be understood as actively caring [51,52], the gerund can of course be turned around to imply objections to others–“do you mind?”

#### Alternative approaches

A few other approaches exist that theoretically could target covert hostility. In research on moral behavior, for example, participants have been asked to distribute tasks with positive, neutral, or even negative consequences between self and others [53,54]. However, the moral option in this line of research of giving the positive task to the other always involved the sacrifice of having to do the less appealing task yourself. The SoMi paradigm involves virtually no sacrifice to the self; being prosocial does not involve high cost, nor does being hostile involve rewards beyond possible effects on the relationship.

Hostility can also be expressed verbally with various degrees of subtlety and covertness; verbal aggression is indeed one of the four components of aggression as identified in the most cited questionnaire on this topic [42], and previous—but not very recent—research has looked at ways to operationalize this [55–57]. But covert or overt, interpretation of verbal messages always depends on language cognition and the corresponding individual differences. The SoMi paradigm does not rely on the filter of language, and mainly appeals to direct preverbal cognition. Instructions are kept to a minimum, and there is no need to train judges in interpreting the hostile content of responses.

Another well-known allocation paradigm that conceptually could be used is SVO, in which participants divide valuable points between self and others. This can be done in an altruistic, prosocial (egalitarian), individualistic, or competitive way. Theoretically, competitive choices could be construed as socially hostile. However, SVO assessments are usually done with general others in mind (i.e., “someone you don’t know and are not likely to meet in the near future”) to establish basic social preferences at trait level [25]. Even though social mindfulness has definite anchors in personality [13], the SoMi paradigm is much more adaptable to the social context, as we have shown here, and thus more sensitive to whom exactly the target is: Is it a friend or a foe?

### Limitations

At this preliminary stage, our findings warrant some caution. All decisions in the current studies were hypothetical, which makes them statements of intention at best. Even though we expect similar findings, it remains to be tested if participants will indeed implement such intentions when confronted with real decisions regarding real interaction partners who are physically present; whether people actually use unmindful choices as a means to express hostility is a question for additional research. Future research could also explicitly address the role of stereotypes or look at reputational concerns as viable alternative explanations; for example, participants could have believed that being unmindful violates social norms, which in turn would reflect poorly on them. Even though we would argue that our findings reflect a more proactive, less defensive stance, it is not unthinkable that people are less likely to violate norms when interacting with friends than with people they will not see again or strongly dislike.

### Concluding Remarks

Soccer is war—but only when not taken too literally. Much more than that, soccer involves healthy competition in which some level of psychological warfare is a legitimate part of the game. Sweeping aggression is the exception, not the rule. Millions of people actually enjoy watching or playing a good game of soccer every week for what it is and should be: A fair and competitive game. In Study 2 we were able to use this competitive environment for its strong ingroup-outgroup setting, however, in which ingroup loyalty was guaranteed to be distinguishable from outgroup competition. In combination with the more general and individually oriented data on friends, strangers, and foes of Study 1, the soccer background provided initial support for the idea that social mindfulness and social hostility are commonly used in relationship maintenance—whether to strengthen or weaken its bonds. While mindful of the restrictions as mentioned earlier, the SoMi paradigm could grow into a useful tool to measure the not always so openly communicated levels of social hostility. After all, the prosocial message of social mindfulness quietly helps define social relationships; it is grease to the wheels of society. Conversely, this makes behavior that can be interpreted as consistently *unmindful* a controlled and effective way to show mild levels of social hostility. Like quietly slipping a few grains of sand in the gears.

## Supporting Information

S1 File Data Study 1. (SAV)

S2 File Data Study 2. (SAV)

S1 Text Supporting information. (PDF)

## References

[1] Sanders E. 'Voetbal is oorlog' is wel degelijk van Rinus Michels [‘Soccer is war’ attributable to Rinus Michels]. NRC Handelsblad. 2005, Mar 11. Available: http://vorige.nrc.nl/krant/article1861181.ece.

[2] Dane Ş, Şekertekin MA. Differences in handedness and scores of aggressiveness and interpersonal relations of soccer players. Percept Motor Skills 2005;100: 743–746. 16060436 10.2466/pms.100.3.743-746

[3] Bredemeier BJ, Weiss MR, Shields DL, Cooper B. The relationship of sport involvement with children’s moral reasoning and aggression tendencies. J Sport Psychol 1986;8(4): 304–318.

[4] Chantal Y, Robin P, Vernat JP, Bernache-Assollant I. Motivation, sportspersonship, and athletic aggression: A mediational analysis. Psychol Sport Exerc, 2005;6: 233–249. 10.1016/j.psychsport.2003.10.010

[5] Tedeschi JT, Felson RB. Violence, aggression, and coercive actions. Washington: American Psychological Association; 1994 10.1037/10160-000

[6] Cuddy AJ, Fiske ST, Glick P. The BIAS map: behaviors from intergroup affect and stereotypes. J Pers Soc Psychol 2007;92: 631–648. 10.1037/0022-3514.92.4.631 17469949

[7] Cuddy AJ, Fiske ST, Glick P. Warmth and competence as universal dimensions of social perception: The stereotype content model and the BIAS map. Adv Exp Soc Psychol 2008;40: 61–149. 10.1016/S0065-2601(07)00002-0

[8] Smith TW. Concepts and methods in the study of anger, hostility, and health In: Siegman AW, Smith TW, editors. Anger, hostility and the heart. Hillsdale: Lawrence Erlbaum; 1994 pp. 23–42.

[9] Elias N. The civilizing process.Vol. 2 transl. Jephcott E.. New York: Pantheon Books; 1982.

[10] Lieberman JD, Solomon S, Greenberg J, McGregor HA. A hot new way to measure aggression: Hot sauce allocation. Aggress Behav 1999;25: 331–348. 10.1002/(SICI)1098-2337(1999)25:5<331::AID-AB2>3.0.CO;2-1

[11] Bushman BJ, Baumeister RF. Threatened egotism, narcissism, self-esteem, and direct and displaced aggression: Does self-love or self-hate lead to violence? J Pers Soc Psychol 1998;75: 219–229. 10.1037/0022-3514.75.1.219 9686460

[12] Ritter D, Eslea M. Hot sauce, toy guns, and graffiti: A critical account of current laboratory aggression paradigms. Aggress Behav 2005;31: 407–419. 10.1002/ab.20066

[13] Van Doesum NJ, Van Lange DAW, Van Lange PAM. Social mindfulness: Skill and will to navigate the social world. J Pers Soc Psychol 2013;105: 86–103. 10.1037/a0032540 23647176

[14] Van Lange PAM, Van Doesum NJ. Social mindfulness and social hostility. Curr Opin Behav Sci 2015;3:18–24. 10.1016/j.cobeha.2014.12.009

[15] Chernyak N, Kushnir T. Giving preschoolers choice increases sharing behavior. Psychol Sci 2013;24: 1971–1979. 10.1177/0956797613482335 23955355

[16] Bown NJ, Read D, Summers B. The lure of choice. J Behav Decis Mak 2003;16: 297–308. 10.1002/bdm.447

[17] Geers AL, Rose JP, Fowler SL, Rasinski HM, Brown JA, Helfer SG. Why does choice enhance treatment effectiveness? Using placebo treatments to demonstrate the role of personal control. J Pers Soc Psychol 2013;105: 549–566. 10.1037/a0034005 23915042

[18] Leotti LA, Iyengar SS, Ochsner KN. Born to choose: The origin and value of the need for control. Trends Cogn Sci 2010;14: 457–463. 10.1016/j.tics.2010.08.001 20817592 PMC2944661

[19] Pierce JR, Kilduff GJ, Galinsky AD, Sivanathan N. From glue to gasoline: How competition turns perspective takers unethical. Psychol Sci 2013;24: 1986–1994. 10.1177/0956797613482144 23955353

[20] Gurtman MB. Trust, distrust, and interpersonal problems: A circumplex analysis. J Pers Soc Psychol 1992;62: 989–1002. 10.1037/0022-3514.62.6.989 1619552

[21] Lewicki RJ, McAllister DJ, Bies RJ. Trust and distrust: New relationships and realities. Acad Manage Rev 1998; 23, 438–458. 10.5465/AMR.1998.926620

[22] Marková I, Gillespie A. Trust and distrust: Sociocultural perspectives. Charlotte, NC: IAP; 2008.

[23] Verburgh L, Scherder EJA, Van Lange PAM, Oosterlaan J. Executive functioning in highly talented soccer players. PLoS One 2014 3 14: e91254 10.1371/journal.pone.0091254 PMC3954684 24632735

[24] Messick DM. Alternative logics for decision making in social settings. J Econ Behav Organ 1999;39: 11–28. 10.1016/S0167-2681(99)00023-2

[25] Murphy RO, Ackermann KA. Social value orientation: Theoretical and measurement issues in the study of social preferences. Pers Soc Psychol Rev 2014;18: 13–41. 10.1177/1088868313501745 24065346

[26] Van Lange PAM. The pursuit of joint outcomes and equalities in outcomes: An integrative model of social value orientation. J Pers Soc Psychol 1999;77: 337–349. 10.1037/0022-3514.77.2.337

[27] Van Lange PAM, Balliet DP, Parks CD, Van Vugt M. Social dilemmas: The psychology of human cooperation. Oxford: Oxford University Press; 2014.

[28] Harbaugh WT, Mayr U, Burghart DR. Neural responses to taxation and voluntary giving reveal motives for charitable donations. Science 2007;316: 1622–1625. 10.1126/science.1140738 17569866

[29] Van Lange PAM, Bekkers R, Schuyt TNM, Van Vugt M. From games to giving: Social value orientation predicts donations to noble causes. Basic Appl Soc Psych 2007;29: 375–384. 10.1080/01973530701665223

[30] Wilson J. Volunteering. Annu Rev Sociol 2000;26: 215–240. Available: http://www.jstor.org/stable/22344325.

[31] Bandura A. Toward a psychology of human agency. Perspect Psychol Sci 2006;1: 164–180. 10.1111/j.1745-6916.2006.00011.x 26151469

[32] Van Prooijen JW, Ståhl T, Eek D, Van Lange PAM. Injustice for all or just for me? Social value orientation predicts responses to own versus other’s procedures. Pers Soc Psychol Bull 2012;38: 1247–1258. 10.1177/0146167212448826 22700243

[33] Van Prooijen JW, Zwenk F. Self-construal level and voice procedures: The individual self as psychological basis for procedural fairness effects. J Exp Soc Psychol 2009;45: 392–397. 10.1016/j.jesp.2008.10.008

[34] Anderson CA, Bushman BJ. Human aggression. Annu Rev Psychol 2001;53: 27–51. 10.1146/annurev.psych.53.100901.135231 11752478

[35] Shaver PR, Mikulincer ME. Introduction In: Shaver PR, Mikulincer ME, editors. Human aggression and violence: Causes, manifestations, and consequences. Washington: American Psychological Association; 2011 pp. 3–11.

[36] Arriaga XB, Schkeryantz EL. Intimate relationships and personal distress. The invisible harm of psychological aggression. Pers Soc Psychol Bull 2015;41: 1332–1344. 10.1177/0146167215594123 26178256

[37] Barefoot JC. Developments in the measurement of hostility In: Friedman HS, editor. Hostility, coping & health. Washington: American Psychological Association; 1992 pp. 13–31.

[38] Crockett MJ, Kurth-Nelson Z, Siegel JZ, Dayan P, Dolan RJ. Harm to others outweighs harm to self in moral decision making. PNAS 2014;111: 17320–17325. 10.1073/pnas.1408988111 25404350 PMC4260587

[39] DeWall CN, Finkel EJ, Lambert NM, Slotter EB, Bodenhausen GV, Pond RS, et al The voodoo doll task: Introducing and validating a novel method for studying aggressive inclinations. Aggress Behav 2013;39: 419–439. 10.1002/ab.21496 23878068

[40] Weingart LR, Behfar KJ, Bendersky C, Todorova G, Jehn KA. The directness and oppositional intensity of conflict expression. Acad Manage Rev 2015;40: 235–262. 10.5465/amr.2013.0124

[41] Buss AH, Durkee A. An inventory for assessing different kinds of hostility. J Consult Psychol 1957;21: 343–49. 10.1037/h0046900 13463189

[42] Buss AH, Perry M. The aggression questionnaire. J Pers Soc Psychol 1992;63: 452–459. 10.1037/0022-3514.63.3.452 1403624

[43] Dodge KA, Malone PS, Lansford JE, Sorbring E, Skinner AT, Tapanya S, et al Hostile attributional bias and aggressive behavior in global context. PNAS 2015;112: 9310–9315. 10.1073/pnas.1418572112 26170281 PMC4522743

[44] Follingstad DR, Coyne S, Gambone L. A representative measure of psychological aggression and its severity. Violence Vict 2005;20: 25–38. 10.1891/0886-6708.2005.20.1.25 16047933

[45] Isberg L. Anger, aggressive behavior, and athletic performance In: Hanin YL, editor, Emotions in sport. Champaign: Human Kinetics; 2000 pp. 113–133.

[46] Dodge KA, Bates JE, Pettit GS. Mechanisms in the cycle of violence. Science 1990;250: 1678–1683. 10.1126/science.2270481 2270481

[47] Elson M, Mohseni MR, Breuer J, Scharkow M, Quandt T. Press CRTT to measure aggressive behavior: The unstandardized use of the competitive reaction time task in aggression research. Psychol Assess 2014;26: 419–432. 10.1037/a0035569 24447279

[48] Van Lange PAM, Otten W, De Bruin EMN, Joireman JA. Development of prosocial, individualistic, and competitive orientations: Theory and preliminary evidence. J Pers Soc Psychol 1997;73: 733–746. 9325591 10.1037//0022-3514.73.4.733

[49] Murphy RO, Ackermann KA, Handgraaf MJJ. Measuring social value orientation. Judgm Decis Mak 2011;6: 771–781. 10.2139/ssrn.1804189

[50] Davis M. Measuring individual differences in empathy: Evidence for a multidimensional approach. J Pers Soc Psychol 1983;44: 113–126. 10.1037/0022-3514.44.1.113

[51] Harvey JH, Omarzu J. Minding the close relationship. Pers Soc Psychol Rev 1997;1: 224–240. 10.1207/s15327957pspr0103_3 15659351

[52] Harvey JH, Omarzu J. Minding the close relationship: A theory of relationship enhancement. Cambridge: Cambridge University Press; 2006.

[53] Batson CD, Lishner DA, Carpenter A, Dulin L, Harjusola-Webb S, Stocks EL, et al “… As you would have them do unto you”: Does imagining yourself in the other's place stimulate moral action? Pers Soc Psychol Bull 2003;29: 1190–1201. 10.1177/0146167203254600 15189613

[54] Batson CD, Thompson ER. Why don't moral people act morally? Motivational considerations. Curr Dir Psychol Sci 2001;10: 54–57. 10.1111/1467-8721.00114

[55] Cahoon DD, Edmonds EM. Guns/no guns and the expression of social hostility. Bull Psychon Soc 1984;22(4): 305–308.

[56] Costin F. The scrambled sentence test: A group measure of hostility. Educ Psychol Meas 1969;29: 461–468.

[57] Epstein N, Krakower S. A measure of verbal aggression. Percept Mot Skills, 1974;39(1): 215–223. 4411654 10.2466/pms.1974.39.1.215

