# Supplementary material for: Social Hostility in Soccer and Beyond
Source: PLoS One. 2016 Apr 14;11(4):e0153577. doi: 10.1371/journal.pone.0153577 (PMC4831831; doi:10.1371/journal.pone.0153577)
Supplement: S1 Text — (PDF) [file pone.0153577.s003.pdf]

Social Hostility in Soccer and Beyond

Supporting Information

PlosOne, 2016

Niels J. Van Doesum[1*], Jan-Willem Van Prooijen[1], Lot Verburgh[2], Paul A. M. Van Lange[1]

[1] Department of Experimental and Applied Psychology, VU Amsterdam, Netherlands

[2] Department of Clinical Neuropsychology, VU Amsterdam, Netherlands

[*] Corresponding author

E-mail: n.j.van.doesum@vu.nl (NVD)

Study 1

In the main text we controlled for age in a general linear model pitting condition (friend, stranger, foe) against social mindfulness. Condition revealed the predicted main effect on social mindfulness, $F(2, 248) = 28.45$, $p < .001$, $\eta_p^2 = .19$. Without controlling for age this was $F(2, 249) = 30.48$, $p < .001$, $\eta_p^2 = .20$.

Location Manipulation

Being surrounded by people they know and have certain expectations of (i.e., who are trusted to a certain extent) may do different things to individuals' attention to others than being in unfamiliar surroundings where general attention is already needed to define one's place in the social environment. As a secondary and exploratory hypothesis, we expected that people might be slightly less socially mindful in a familiar than in an unfamiliar environment. We therefore randomly assigned participants to a location condition.

A familiar location (1) was described as: “Let's say that you are sitting together in a familiar café, where you come more often and know at least a couple of people by face and/or name.” An unfamiliar location (2) was introduced as: “Let's say that you are sitting together in an unfamiliar café, where you have never been before and where you don't know or recognize anybody.” However, this manipulation failed, as 37% of the participants did not pass the manipulation check asking to recall the location. For theoretical clarity and conciseness we decided to not report this variable in the main text. Still, as a double check, running a GLM with the remaining 158 participants in a 3 (relationship; friend, stranger, foe) x 2 (location; familiar, unfamiliar) design, and controlling for age, confirmed the significant effect of the relationship condition, $F(2, 153) = 11.37$, $p < .001$, $\eta^2 = 0.11$, versus a non-significant effect for location, $F(1, 153) = 1.25$, $p = .266$, $\eta^2 = 0.01$. There was no significant interaction. Location therefore did not play a role in social hostility or social mindfulness.

Measures

As an exploratory measure, we had added a measure of trust after the main procedure. Interpersonal trust was measured in 3 items, answered on a 7-point scale from *strongly disagree* to *strongly agree*, e.g., "I completely trust the other person," $\alpha = .81$. We also measured the extent to which people felt the other person would trust them, e.g., "The other person completely trusts me," 3 items, $\alpha = .73$ [1]. To explore future expectations regarding the other, we furthermore included a new three item scale asking participants how likely they thought they were to meet the other person in the future; if they thought their choices would impact their future relationship; and whether they expected their choices to influence the image the other person has of them. However, these items proved not to combine into a reliable scale ($\alpha = .23$), and were not analyzed any further. Finally, we asked participants how good they thought the relationship with the other person was (to provide us with an assessment of relationship quality; 1 item). All answers were scored on a 7-point scale.

Results

We explored whether interpersonal trust might mediate the effect of condition (friend, stranger, foe), such that greater social mindfulness would be explained by a higher level of trust in the other. Condition indeed was related to interpersonal trust, in the sense that participants trusted friends more than strangers, but strangers more than foes; $M_{\text{friend}} = 5.29$ ($SD = 1.05$), $M_{\text{stranger}} = 3.11$ ($SD = 1.01$), $M_{\text{foe}} = 1.87$ ($SD = 0.82$), $F(2, 249) = 273.68$, $p < .001$, $\eta^2 = 0.69$. When interpersonal trust was added to the general model, it showed to have a small but significant effect on social mindfulness, $F(1, 247) = 5.57$, $p = .019$, $\eta^2 = 0.01$, whereas the effect of condition decreased in size, $F(2, 247) = 4.21$, $p = .016$, $\eta^2 = 0.03$. Bootstrapping procedures [2] confirmed that the bias corrected confidence intervals did not include zero. This suggests that higher interpersonal trust may help explain why participants were more mindful of their friends, versus socially hostile towards foes.

In a separate regression analysis, relationship quality predicted social mindfulness, *b* = .06, *t*(250) = 7.74, $p < .001$, $R^2 = .19$, in the sense that higher quality was associated with greater social mindfulness; but adding this to the general model reported above did not alter its conclusions, and the effect of this variable on social mindfulness disappeared ($p = .200$). The same was the case for the extent to which participants felt trusted by the other: *b* = .06, *t*(250) = 6.33, $p < .001$, $R^2 = .14$, but not significant ($p = .300$) when controlling for this variable in the general model.

Study 2

Control Group

In the context of our study on social hostility, there was another control group within the subject pool that consisted of 42 sedentary children and adolescents who did not play any sports. This group included a few female participants, however, and ingroup identification was obviously not as strong and identifiable. For the sake of theoretical clarity, the scores of this group were not included in the analyses. However, running the same analysis with these participants included did not structurally alter the pattern and/or the strength of the conclusions.

SoMi Paradigm

We had added two ingroup specific item categories to the original SoMi paradigm (which used 5 categories). Running the same analysis as in the main text, but without the scores on the trials that used these items, revealed similar results: No main effect for control versus experimental round, $F(1, 131) = 0.68$, $p = .411$, $\eta_p^2 = .01$, but a significant interaction between condition (ingroup, outgroup) and the two rounds of the SoMi paradigm, $F(1, 131) = 18.24$, $p < .001$ $\eta_p^2 = .12$. In the experimental (second) round ingroup members ($M_{ingroup}$ = .58, *SD* = .26) had exhibited greater social mindfulness towards one another than outgroup

members ($M_{outgroup}$ = .46, SD = .24), while both significantly differed from their respective baseline scores regarding strangers in opposite directions (higher for ingroup, lower for outgroup), p = .015 and p = .001, respectively.

References

1. Van Lange PAM, Vinkhuyzen, AAE, Posthuma D. Genetic influences are virtually absent for trust. PLoS One 2014 Apr 7: e93880. doi:10.1371/journal.pone.0093880
2. Preacher KJ, Hayes AF. Asymptotic and resampling strategies for assessing and comparing indirect effects in multiple mediator models. Behav Res Methods 2008;40: 879–891. doi:10.3758/BRM.40.3.879
